# Quantifying pharmacologic suppression of cellular senescence: prevention of cellular hypertrophy versus preservation of proliferative potential

**DOI:** 10.18632/aging.100115

**Published:** 2009-12-31

**Authors:** Zoya N. Demidenko, Mikhail V. Blagosklonny

**Affiliations:** Oncotarget, Buffalo, NY 14263, USA; Department of Cell Stress Biology, Roswell Park Cancer Institute, Buffalo, NY 14263

**Keywords:** cellular senescence, cellular hypertrophy, aging-suppression, rapamycin, mTOR

## Abstract

Development of agents that suppress aging (aging suppressants) requires quantification of cellular senescence. Cellular senescence in vitro is characterized by a large cell morphology and permanent loss of proliferative potential. When HT-1080 cells were arrested by p21, they continued to grow exponentially in size and became hypertrophic with a 15-fold increase in the protein content per cell. These changes were mirrored by accumulation of GFP (driven by CMV promoter) per cell, which also served as a marker of cellular hypertrophy. Preservation of proliferative potential (competence) was measured by an increase in live cell number, when p21 was switched off. While modestly decreasing hypertrophy in p21-arresrted cells, rapamycin considerably preserved competence, converting senescence into quiescence. Preservation of proliferative potential (competence) correlated with inhibition of S6 phosphorylation by rapamycin. When p21 was switched off, competent cells, by resuming proliferation, became progressively less hypertrophic. Preservation of proliferative potential is a sensitive and quantitative measure of suppression of mTOR-driven senescence.

## Introduction

In cell culture, cellular senescence is usually defined as a state of irreversible cell cycle arrest [1,2]. Hence, cellular senescence is sometimes confused with growth inhibition. Here we will use the term ‘growth' as an increase in cellular mass, regardless of whether cells proliferate or not. Intriguingly, Ras, MEKeIF-4E and serum, which stimulate growth-promoting pathways, contribute to and facilitate cellular senescence [3-6].  In theory, cellular senescence is caused by inappropriate activation of growth-promoting pathways, when actual growth is impossible [7,8]. In proliferating cells, growth-promoting mTOR (Target of Rapamycin) and MAPK (Mitogen-activated Protein Kinase) pathways drive both cellular mass growth and cell cycle progression. When the cell cycle is blocked by either p21 or p16, growth-stimulation via mTOR leads to cellular senescence [9]. Serum withdrawal, PI-3K, mTOR and MEK inhibitors, all decreased mTOR activity and prevented permanent loss of proliferative potential [10,11].  The term "permanent loss of proliferative potential" means that, even when p21 and p16 were shut off, cells cannot resume proliferation [12]. Inhibitors of mTOR such as rapamycin preserved proliferative potential [9-11]. To avoid confusions, we stress that rapamycin does not stimulate proliferation, does not abrogate cell cycle arrest caused by p21 and does not force cells to by-pass cell cycle arrest. Rapamycin converts senescence (an irreversible condition) into quiescence (a reversible condition). It is still unknown whether rapamycin suppresses senescence in a dose-dependent manner and whether this suppression correlates with the degree of mTOR inhibition.

Another common marker of cell senescence is a large cell morphology (hypertrophy). Cellular hypertrophy is usually measured as a cell diameter. Given that volume (or cell mass) is proportional to the cube of diameter, then the amount of protein per cell (cell mass) may be a more sensitive parameter than cell diameter. For example if diameter is increased 2-fold, cell mass is increased 8-fold. In theory, cell mass could be estimated as an amount of any fluorescent protein such as green fluorescent protein (GFP), expressed by a constitutive viral promoter such as CMV promoter. If the cell cycle is blocked but cells continue to grow in size, then GFP should accumulate. Here we tested this prediction. Independently from our study, a clone of HT-p21 cells, known as p21-9, had been stably transfected with CMV-EGFP [13,14,15] and thus expresses enhanced GFP. We predict that induction of p21 by IPTG should increase GFP per cell, as a marker of cellular hypertrophy. Given cell-doubling time of 20 hours, there should be a 10-14 fold increase in GFP/cell in 3 days. Here, we confirmed this prediction. We further investigated the link between mTOR activity, cellular hypertrophy and loss of proliferative potential.  We found that preservation of proliferative (competence) was the most sensitive marker of mTOR inhibition, easily detectable even at concentrations of rapamycin when inhibition of mTOR was marginal.

## Results

### Exponential mass-growth precedes senescence

**Figure 1.**
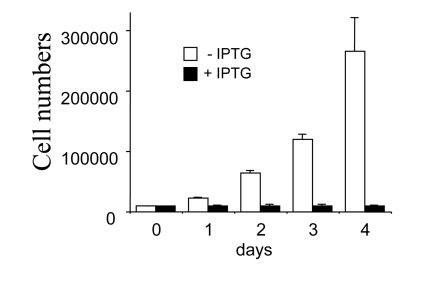
Inhibition of cell proliferation by IPTG. Closed bars: HT-p21 cells were treated with IPTG (+IPTG). Cells do not proliferate. Open bars: Untreated HT-p21 cells. Exponentially proliferating cells. Cells were counted daily.

**Figure 2.**
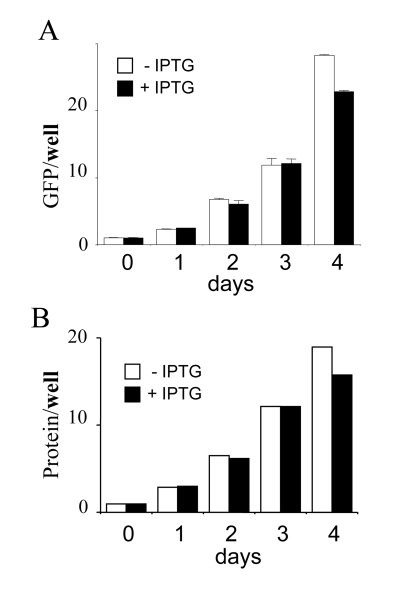
Total cellular mass growth during senescence induction. HT-p21 cells were grown in 60 mm wells and soluble protein and GFP were measured daily. Closed bars: HT-p21 cells were treated with IPTG (+IPTG). Open bars: Untreated HT-p21 cells (-IPTG). In both proliferating (-IPTG) and non-proliferating (+IPTG) conditions, protein per well and GFP per well were increasing.  In panel **B**, protein was measured in duplicate and shown without standard deviations, therefore statistical difference between –IPTG and + IPTG should not be considered. The panel simply illustrates exponential growth in both conditions.

**Figure 3.**
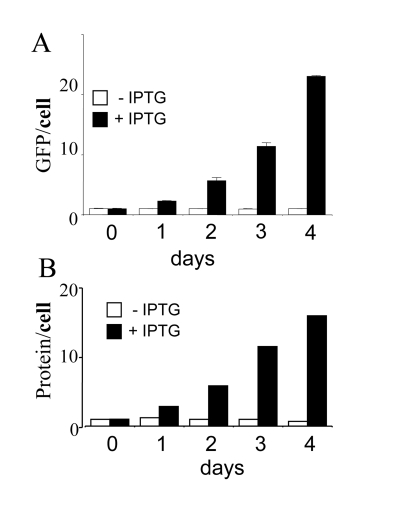
Cellular hypertrophy during senescence induction. HT-p21 cells were grown in 60 mm wells and cell numbers, soluble protein and GFP were measured daily. Closed bars: HT-p21 cells were treated with IPTG (+IPTG). Open bars: Untreated HT-p21 cells (-IPTG). Protein per cell and GFP per cell were constant in proliferating (-IPTG) cells.  Protein per cell and GFP per cell increased exponentially in non-proliferating (+IPTG) cells.

A number of proliferating cells increased exponentially (with a doubling time 20-24 h). As previously described, induction of p21 by IPTG caused G1 and G2 arrest [1,4,5], completely blocking cell proliferation (Figure 1). p21-arrested cells continued to grow in size, becoming hypertrophic. Since the cells contained CMV-driven EGFP, we measured both protein and GFP. Per well, amounts of GFP and protein were increased almost exponentially with or without IPTG (Figure 2). Per cell, amounts of GFP and protein were increased only for IPTG-treated (non-dividing) cells (Figure 3).  For proliferating cells (no IPTG), GFP per cell and protein per cell remained constant (Figure 3), because mass growth was balanced by cell division. In contrast, in IPTG-treated cells, protein/cell and GFP/cell increased almost exponentially for 3 days (Figure 3). During induction of senescence by IPTG, cellular mass continued to increase but was not balanced by cell division.  In all cases, protein and GFP correlated (Figure 3), making GFP per cell a convenient marker of cellular hypertrophy.

Although that was not the goal of our study, our data can explain how induction of p21 can induce GFP without trans-activating CMV promoter: by inhibiting cell cycle without inhibiting cell growth. Furthermore, the notion that GFP per cell is a marker of hypertrophy yields 2 predictions. First, mutant p21 that cannot bind CDKs and thus cannot arrest cell cycle will not induce GFP. Second, antihypertrophic agents such as rapamycin will reduce GFP per cell without abrogating cell cycle arrest.

### Dose dependent suppression of cellular hypertrophy

**Figure 4.**
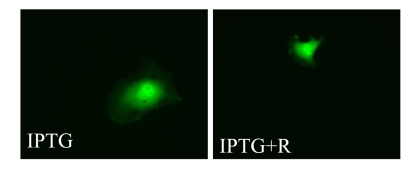
Visualization of cellular hypertrophy. HT-p21 cells express enhanced green fluorescent protein (GFP) under the constitutive viral CMV promoter. Expression of GFP per cell is a marker of cellular hypertrophy. Low cell density - 2 thousand cells were plated in 100 mm dish and treated with either IPTG or IPTG + Rapamycin.

**Figure 5.**
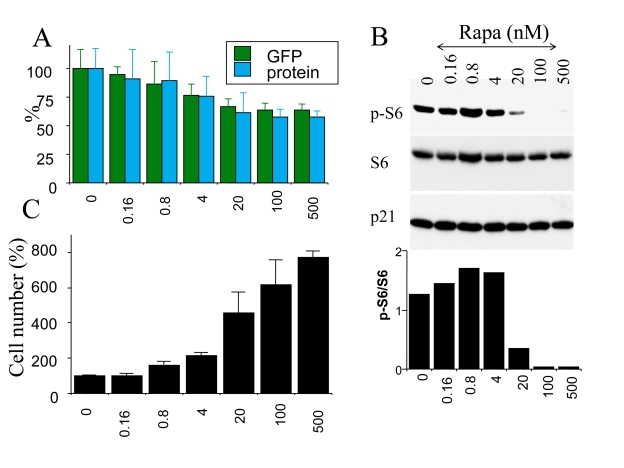
Correlation between S6 phosphorylation, hypertrophy and loss of proliferative potential in senescent cells. HT-p21 cells were plated in 6 well plates and treated with IPTG plus the increasing concentrations of rapamycin (from 0.16 to 500 nM). At concentration 0, cells were treated with IPTG alone. (**A**) Cellular hypertrophy: protein and GFP. After 3 days, soluble protein and GFP were measured per well. [Note: in non-proliferating cells, protein/well is a measure of protein/cells]. Results are shown as percent of IPTG alone (0) without rapamycin. (**B**) After 3 days, cells were lysed and immunobloted for p-S6, S6 and p21. (**C**) PC: preservation of proliferative competence. After 3 days, cells were washed to remove IPTG and RAPA. Cells were incubated for additional 5 days in the fresh medium and then were counted. Results are shown as percent of IPTG alone (0) without rapamycin.

We next investigated the effects of rapamycin on hypertrophy of senescent cells. Cells were induced to senesce by IPTG in the presence (+R) or the absence of rapamycin. On days 3 and 5 effects of rapamycin on cellular hypertrophy were evaluated. By microscopy, the anti-hypertrophic effect of rapamycin was the most evident at low cell densities (such as 1000 cells per 60-mm dish) because there was a sufficient space for IPTG-treated cells to grow in size in the absence of rapamycin (Figure 4). However, we could not reliably measure protein levels at such low cell densities. At regular cell densities, rapamycin (500 nM) reduced cellular hypertrophy by 30% -40% (Figure 5A and data not shown). Two markers of hypertrophy (protein/cell and GFP/cell) correlated (Figure 5A). The anti-hypertrophic effect of rapamycin was not statistically significant at concentrations of rapamycin below 20 nM. At first, this was puzzling given that rapamycin inhibits the mTOR pathway at low concentrations in many cell types. Therefore, we investigated a dose response of mTOR inhibition by measuring S6 phosphorylation, a marker of mTOR activity. In agreement with anti-hypertrophic effects, rapamycin inhibited S6 phosphorylation at concentrations 20 nM or higher, achieving maximal effects at 100 nM-500 nM (Figure 5 B). Thus, inhibition of S6 phosphorylation and inhibition of hypertrophy correlated, explaining the requirements of high concentration (100-500 nM) of rapamycin for anti-hypertrophic effects in this particular cell line.

### Dose-dependent preservation of cellular competence

Rapamycin preserves proliferative potential in arrested cell, meaning that cells can successfully divide when the arrest is lifted. But rapamycin does not induce proliferation and in contrast can cause quiescence (in some cell types). To clearly distinguish the potential to proliferate (competence) and actual proliferation, we introduce terms competence and incompetence (permanent loss of proliferative potential associated with cellular senescence). In HT-1080 cells, rapamycin preserves competence during cell cycle arrest caused by [10]. Unlike senescent cells, quiescent cells are competent.

We have demonstrated previously that rapamycin preserved cellular competence (the ability to proliferate after p21 is switched off) in IPTG-arrested HT-p21 cells [10]. We performed these experiments using rapamycin at concentration 500 nM [10], which completely inhibited S6 phosphorylation. Here we determined whether preservation of competence (PC) correlated with inhibition of S6 phosphorylation and the anti-hypertrophic effect of rapamycin. Cells were treated with IPTG and increasing concentrations of rapamycin ranging from 0 to 500 nM (Figure 5 C). After 3 days, IPTG was washed out, thus allowing the cells to proliferate, and after another 5 days cells were counted. As expected, the IPTG-treated cells became incompetent, whereas rapamycin suppressed incompetence (Figure 5 C). Remarkably, preservation of competence was detectable at lower concentrations of rapamycin than those that inhibited either S6 phosphorylation or cellular hypertrophy. In part, such a higher sensitivity of a PC-test compared with inhibition of hypertrophy may be due to the relative magnitudes of the effects (30% inhibition of hypertrophy versus 800% PC). Perhaps even a transient inhibition of mTOR (missed by immunoblot) detectably increased competence. Consistent with this explanation, even when rapamycin was added with delay, preservation of competence was detectable [10].

### Exponential proliferation of competent cells

**Figure 6.**
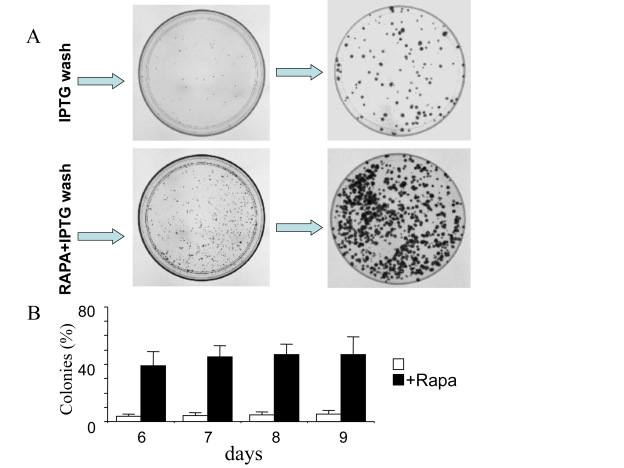
Clonal proliferation of competent cells. HT-p16 cells were plated in 100-mm plates. The next day, 50 μM IPTG with or without rapamycin, if indicated (RAPA), was added. After 3 days, the plates were washed to remove IPTG and RAPA. (**A**) Photographs. Upper panel: On days 5 and 8 (after IPTG removal), plates were fixed, stained and photographed. Lower panel: On days 5 and 8 (after IPTG removal), plates were fixed, stained and photographed.  (**B**) Number of colonies. On days 6, 7, 8 and 9 (after IPTG removal), plates were fixed, stained and photographed. The number of colonies was counted and results are shown as percent of plated cells in log-scale.

**Figure 7.**
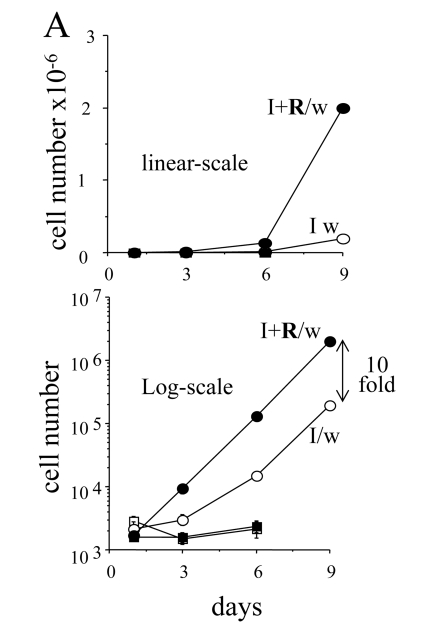
The dynamics of cell numbers. 500 HT-p21 cells were plated in 12 well plates. On the next day, either IPTG alone (I) or IPTG plus rapamycin (I+R) were added. After 3 days, plates were washed (I/w and I+R/w) or left unwashed. Cells were counted at days 1, 3, 6 and 9. Upper panel: linear-scale. Lower panel: log-scale. Open and closed squares: IPTG and IPTG plus Rapa, respectively. Open and closed circles: IPTG washed (I/w) and IPTG plus Rapa washed (I+R/w), respectively. In the presence of IPTG (open squares) and IPTG plus rapamycin (closed squares), the cells did not proliferate.

In the presence of IPTG (with or without rapamycin), the cells did not proliferate and did not form colonies. When IPTG was washed out, 3-5% cells remained competent even without rapamycin [10] and Figure 6. Colonies grew in size, while the number of colonies was almost unchanged (Figure 6). Rapamycin increased a number of colonies (a number of competent cells) almost 10- fold. We further compared the proliferative quality of competent cells remained after treatment with IPTG either without or with rapamycin (I/w and I+R/w, respectively). In I/w and I+R/w conditions, the number of cells started to increase exponentially after 1 day and 3 days, respectively (Figure 7). After 6 days, both curves (I/w and I+R/w) became parallel.  The curve "I+R/w" was just shifted to the right on approximately 3 days (Figure 7). This corresponded to a 10-fold difference in an initial number of competent cells, if their doubling time was around one day. Noteworthy, this also corresponds to the initial difference in the number of competent cells as determined by colony formation (Figure 6). Also, both in I/w and I+R/w conditions, doubling time of the competent cells was around 20-24 hours, similar to the proliferative rate of the untreated cells.

### Reversal of hypertrophy during proliferation of competent cells

**Figure 8.**
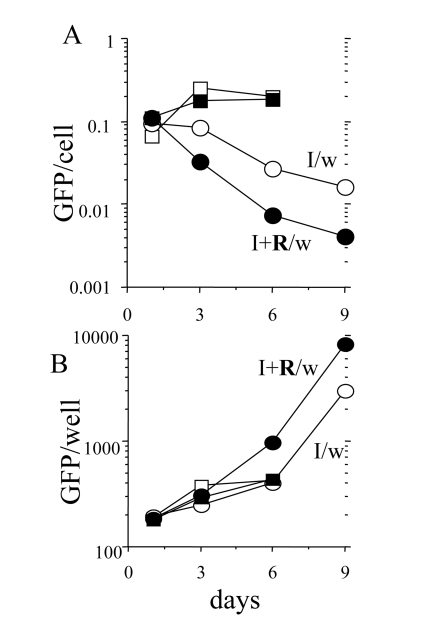
Loss of hypertrophy during proliferation of competent cells. 500 HT-p21 cells were plated in 12 well plates. The next day, either IPTG alone or IPTG plus rapamycin were added. After 3 days, plates were washed (I/w and I+R/w) or left unwashed. GFP per well was measured and cells were counted at days 1, 3, 6 and 9. GFP per cell was calculated (upper panel). Results are shown in arbitrary units (M±m). Open and closed squares: IPTG and IPTG plus Rapa, respectively. Open and closed circles: IPTG washed (I/w) and IPTG plus Rapa washed (I+R/w), respectively. When cells resumed exponential proliferation, GFP per cell dropped to normal levels.  Due to robust proliferation, there was an increase of GFP per well.

**Figure 9.**
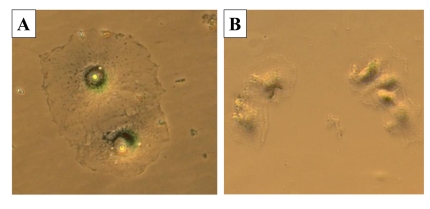
The morphology of cells during recovery. 500 HT-p21 cells were plated in 12 well plates. The next day, IPTG (**A**) or IPTG plus rapamycin (**B**) was added. After 3 days, plates were washed and microphotographs were taken after additional 3 days. Cells were stained for beta-Gal. A: I/w; B:  I+R/w.

**Figure 10.**
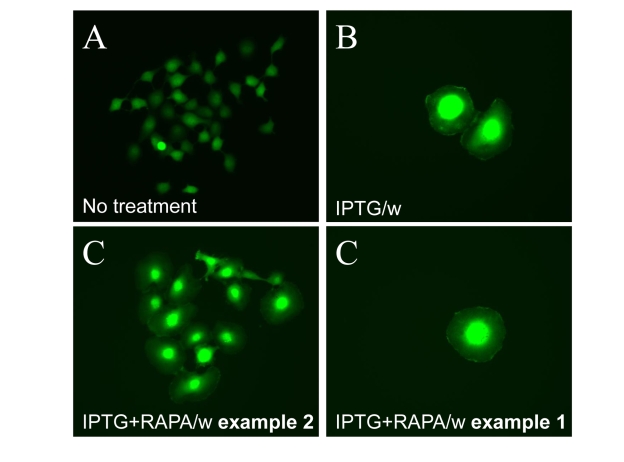
Visualization of loss of hypertrophy during proliferation of competent cells. 500 HT-p21 cells (**A**) were treated with IPTG (**B**) or IPTG plus rapamycin (**C**), as indicated, or left untreated. After 3 days, plates were washed and incubated without drugs to allow proliferation. (**A**) Normal size of proliferating cells. (**B**) Cellular hypertrophy of senescent cells. (**C**) Example 1. Clonal proliferation of competent cells results in loss of hypertrophy. (**C**) Example 2. Cells that remained arrested remained hypertrophic.

Rapamycin decreased cellular hypertrophy approximately 30% in IPTG treated cells (Figure 5A). When IPTG and rapamycin were washed out, there was a lag period about 24-30 hrs for competent cells to undergo first division (supplementary movie will be available at). During the lag period, cells grew in size, because rapamycin was washed out. Consequently, as measured by GFP per cell (Figure 8A), rapamycin-treated cells reached the size of the cells treated with IPTG alone (Figure 8A: I/w and I+R/w at day one). Similarly, as measured by protein per cell, the cells treated with IPTG plus rapamycin become fully hypertrophic at day one after wash (data not shown). Despite regaining hypertrophy, IPTG+rapamycin-treated cells remained competent (Figures 6, 7). This indicates that hypertrophy was not a cause of proliferative incompetence in IPTG-treated cells. When competent cells divided, GFP per cell decreased (Figure 8 B). In agreement, there was a marked difference in cell morphology of typical cells in both conditions (Figure 9). Under I/w conditions, most of the cells were still large and flat, expressing beta-Gal staining. Under I+R/w conditions, predominant cells were with a small-cell morphology and beta-Gal-negative. These cells formed colonies, indicating that they acquired non-senescent morphology due to proliferation (Figure 10 C, example 1). In contrast, senescent cells that did not resume proliferation remained large (Figure 10 C, example 2). Competent cells, while proliferating and forming colonies, became smaller in size (Figure 10 C, example 1). Eventually, the average cell size dropped to normal levels under I+R/w conditions, coincident with a decrease in both the amount of protein/cell and GFP/cell coincided (Supplemental Figure  2), indicating that both are markers of cellular hypertrophy. Despite reversal of hypertrophy and a drop in GFP/cell, the amount of total GFP and protein per well increased due to cell proliferation (Figure 8 B and data not shown).

## Discussion

Acting in concert, three conditions can contribute to cellular hypertrophy: cell cycle arrest, continuous protein synthesis and insufficient autophagy. When the cell cycle was blocked by p21, HT-p21 cells grew in size almost exponentially for 3 days, eventually becoming senescent. In parallel with protein content, the amount of GFP (driven by the CMV promoter) per cell was increased up to 15-20-fold in senescent cells, an increase that may be a marker of cellular hypertrophy.

Why cells did not grow in size indefinitely while turning into senescent cells? First, cellular growth may become counter-balanced by autophagy. This is likely, given the increase in beta-Gal staining and vacuolarization in senescent cells and the recent finding that autophagy is activated several days after senescence induction, coincident with spontaneous deactivation of the PI-3K/mTOR pathway [16]. We also observed dephosphorylation of S6, when IPTG-treated cells became terminally-senescent (MS in preparation). Also, senescent cells may become compensatory insensitive to growth factors.

Rapamycin modestly (30-40%) suppressed cellular hypertrophy and dramatically (10-fold) increased the number of competent (for proliferation) cells.  When competent cells were released from p21-induced block, they first grow in size for one day (before division) and then divided. This indicates that hypertrophy per se does not preclude normal mitosis. While dividing and proliferating, such cells became progressively smaller. This recovery phase is a mirror image of the senescence-induction phase, in which cells grow without division.

How can we explain preservation of mitotic competence by rapamycin? This unlikely results from the anti-hypertrophic effect of rapamycin, given that after rapamycin removal competent cells ‘catch up' in size with other cells. We suggest that mitotic incompetence is not caused by hypertrophy but rather hypertrophy and incompetence are independent hallmarks of cellular aging. We hypothesize that mitotic incompetence may result from cellular hyper-activation during cell cycle arrest. Activated mTOR and MAPK pathways may force cell cycle progression despite p21-induced arrest, causing abortive S-phase entry. In fact, cyclin D1 is highly elevated in senescent cells [9] and Rb is depleted [17]. In principle elevation of cyclins and depletion of Rb may allow p21-arrested cells to enter S-phase, thus damaging the cell. Perhaps, premature cell cycle progression and mitotic incompetence are two sides of the same coin: overactivation of growth promoting and mitogen-activated pathways during cell cycle arrest. Then unscheduled S phase re-entry might be preventable by rapamycin. This hypothesis is under investigation. Noteworthy, rapamycin blocks pseudo-DNA damage response, associated with cellular overactivation [18].  Another hallmark of cellular over-activation in senescent cells is hypersecretory and pro-inflammatory phenotype, characterized by production of cytokins, mitogens and proteases [19-26]. Needles to say, rapamycin is an anti-inflammatory drug and is labeled for use (at high doses) as immunosuppressant in the clinic. It was suggested that rapamycin as an anti-aging drug will extend healthy and maximal lifespan in humans [27-31].

## Materials and methods

Cell lines and reagents .  In HT-p21 cells, p21 expression can be turned on or off using isopropyl--thio-galactosidase (IPTG) [14,15]. HT-p21 cells were cultured in DMEM medium supplemented with FC2 serum. Rapamycin was obtained from LC Laboratories and dissolved in DMSO as 2 mM solution and was used at final concentration of 500 nM, unless otherwise indicated. IPTG and FC2 were obtained from Sigma-Aldrich (St. Louis, MO). IPTG was dissolved in water as 50 mg/ml stock solution and used in cell culture at final concentration of 50 μg/ml.

Immunoblot analysis . Cells were lysed and soluble proteins were harvested as previously described [9]. Immunoblot analysis was performed using mouse monoclonal anti-p21, mouse monoclonal anti-phospho-S6 Ser240/244 (Cell Signaling, MA, USA), rabbit polyclonal anti-S6 (Cell Signaling, MA, USA) and mouse monoclonal anti-tubulin Ab as previously described [9].

Cell counting. Cells were counted on a Coulter Z1 cell counter (Hialeah, FL).

Colony formation assay . Two thousand HT-p21 cells were plated per 100 mm dishes. On the next day, cells were treated with 50 μg/ml IPTG and/or 500 nM rapamycin, as indicated. After 3 days, the medium was removed; cells were washed and cultivated in the fresh medium. When colonies become visible, plates were fixed and stained with 0.1% crystal violet (Sigma).  Plates were photographed and the number of colonies were determined as previously described [9].

SA-β-Gal staining . Cells were fixed for 5 min in β-galactosidase fixative (2 % formaldehyde; 0.2% glutaraldehyde in PBS), and washed in PBS and stained in β-galactosidase solution (1 mg/ml 5-bromo-4-chloro-3-indolyl-beta-gal (X-gal) in 5 mM potassium ferricyamide, 5 mM potassium ferrocyamide, 2 mM MgCl_2_ in PBS) at 37 ºC until beta-Gal staining become visible in either experiment or control plates. Thereafter, cells were washed in PBS, and the number of -galactosidase activity-positive cells (blue staining) were counted under bright field illumination.

## Supplemental figures

**Figure S1.**
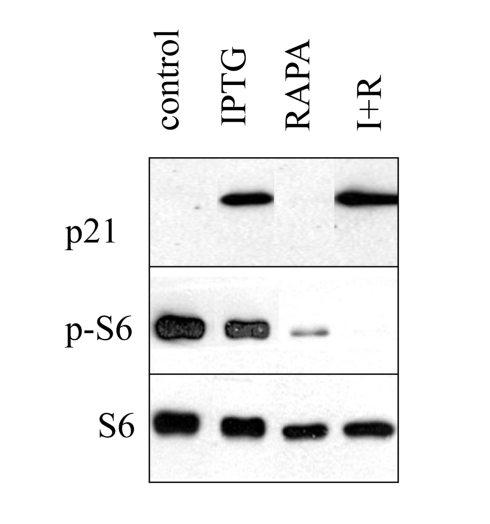
Induction of p21 by IPTG. HT-p21 cells were plated in 6 well plates and treated with IPTG with or without rapamycin as indicated. The next day, cells were lysed and immunoblot for p-S6, S6 and p21 was performed as described in Methods. IPTG dramatically induced p21, without affecting S6 phosphorylation, whereas rapamycin inhibited S6 phosphorylation, without affecting p21 induction.

**Figure S2.**
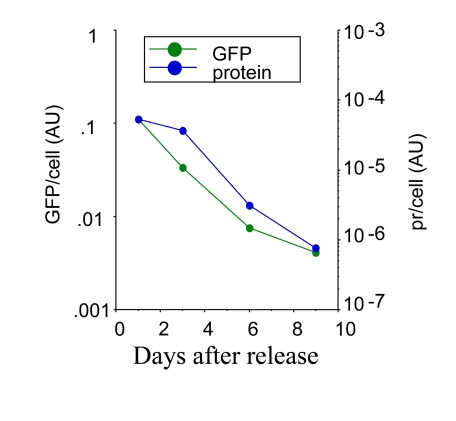
Loss of hypertrophy following release. HT-p21 cells were treated with IPTG plus 500 nM rapamycin for 3 days. Then the cells were washed and the cells were incubated in the fresh medium without drugs. At indicated days, soluble protein, GFP and cell numbers were measured per well. Protein (pr) per cell and GFP per cell were calculated and plotted in arbitrary units.
